# *Drosophila* microRNA-34 Impairs Axon Pruning of Mushroom Body γ Neurons by Downregulating the Expression of Ecdysone Receptor

**DOI:** 10.1038/srep39141

**Published:** 2016-12-23

**Authors:** Yen-Wei Lai, Sao-Yu Chu, Jia-Yi Wei, Chu-Ya Cheng, Jian-Chiuan Li, Po-Lin Chen, Chun-Hong Chen, Hung-Hsiang Yu

**Affiliations:** 1 Institute of Cellular and Organismic Biology, Academia Sinica, Taipei, Taiwan; 2 Institute of Molecular and Genomic Medicine, National Health Research Institutes, Miaoli County, Taiwan; 3 Institute of Molecular and Cellular Biology, College of Life Science, National Taiwan University, Taipei, Taiwan

## Abstract

MicroRNA-34 (miR-34) is crucial for preventing chronic large-scale neurite degeneration in the aged brain of *Drosophila melanogaster*. Here we investigated the role of miR-34 in two other types of large-scale axon degeneration in *Drosophila*: axotomy-induced axon degeneration in olfactory sensory neurons (OSNs) and developmentally related axon pruning in mushroom body (MB) neurons. Ectopically overexpressed miR-34 did not inhibit axon degeneration in OSNs following axotomy, whereas ectopically overexpressed miR-34 in differentiated MB neurons impaired γ axon pruning. Intriguingly, the miR-34-induced γ axon pruning defect resulted from downregulating the expression of ecdysone receptor B1 (EcR-B1) in differentiated MB γ neurons. Notably, the separate overexpression of EcR-B1 or a transforming growth factor- β receptor Baboon, whose activation can upregulate the EcR-B1 expression, in MB neurons rescued the miR-34-induced γ axon pruning phenotype. Future investigations of miR-34 targets that regulate the expression of EcR-B1 in MB γ neurons are warranted to elucidate pathways that regulate axon pruning, and to provide insight into mechanisms that control large-scale axon degeneration in the nervous system.

During development, neurons are assembled into functional neural pathways through a series of tightly regulated steps involving neurogenesis, neural fate specification, the extension of axons and dendrites, the morphogenesis of axons and dendrites to initiate synaptogenesis, and the culling out of excessive and inappropriate synapses^1^. When neurons of the central nervous system (CNS) are damaged as a result of injury or age-associated processes, degenerative process is initiated that can give rise to neurological diseases^1^. Elucidating the molecular mechanisms underlying the various steps involved in neural development might provide insights into the processes that contribute to the maintenance and eventual deterioration of the nervous system over an animal’s lifespan.

MicroRNAs (miRNAs) are a phylogenetically-conserved class of short single-stranded noncoding RNAs that regulate the expression of sets of genes at the post-transcriptional level^2^,^3^,^4^. Accumulating evidence shows that miRNAs play essential roles in the development, physiology, and degeneration of the nervous system^2^,^3^,^4^. In the developing brain, miR-9 and miR-124 are crucial for neurogenesis and neuronal differentiation^4^, whereas miR-34 has been shown to regulate tau expression in cultured human neuroblastoma cells (and changes in the level of tau expression are known to link to the pathology of Alzheimer’s disease)^5^,^6^. Interestingly, *mir-34* loss-of-function (LOF) mutants exhibit the enhancement of the formation of sporadic vacuoles in the aged fly brain, which is characteristic of large-scale neurite degeneration in chronic neural deterioration^7^. In aged adult flies, elevated miR-34 expression reduces the inclusion of stress chaperones, including Hsp70/Hsc70, and inhibits the expression of Ecdysone-induced protein 74EF (Eip74EF), which partially prevents the progression of adult-onset chronic neurodegeneration^7^. However, by the fact that miRNAs often regulate the expression of multiple genes and varied biological processes, it is unclear whether other miR-34-regulated genes are involved in age-related large-scale neurite degeneration and whether similar molecular mechanisms are also shared in different types of large-scale neurite degeneration.

In *Drosophila*, two other types of large-scale neurite degeneration, including injury-induced axon degeneration in olfactory sensory neurons (OSNs) and developmentally related large-scale axon pruning in mushroom body (MB) γ neurons, have been investigated extensively for their underlying molecular mechanisms^8^,^9^. For example, the *Drosophila* sterile α/Armadillo/Toll-interleukin receptor homology domain protein (dSarm) is required for the acceleration of axon degeneration following axotomy of OSNs^10^. Although the mechanism by which dSarm promotes axon degeneration remains unclear, Toll-interleukin 1 repeat protein, the homolog of dSarm in *Caenorhabditis elegans*, has been shown to function downstream of voltage-gated calcium channel and calmodulin-dependent protein kinase II, thereby representing a mechanistic link between the dramatic increase in Ca^2+^-mediated signaling in severed axons and the initiation of axon degeneration^11^,^12^,^13^. Moreover, the Highwire E3 ubiquitin ligase has been shown to promote axon degeneration in OSNs by fine tuning the expression level of the NAD^+^ biosynthetic enzyme, nicotinamide mononucleotide adenylyl transferase, a crucial component for protecting the degeneration of severed axons through a gain-of-function mechanism^14^,^15^,^16^,^17^. In contrast, axon pruning is initiated in MB neurons during the early pupal stage, in which larval-specific dorsal and medial γ lobes are eliminated, and adult-specific γ lobes are formed during the mid-pupal stage^18^. The insect molting hormone, 20-hydroxyecdysone (ecdysone), and its heterodimeric receptors, consisting of ultraspiracle (USP) and ecdysone receptor (EcR), contribute to the regulation of axon pruning of MB γ neurons^19^. The transforming growth factor (TGF)- β receptor, Baboon (Babo), and its downstream target molecule, Smad on X (Smox), also influence axon pruning of MB γ neurons by regulating the expression of the EcR isoform, EcR-B1^20^. However, it remains unclear whether miRNAs are also involved in these two types of axon degenerative processes.

In this study, we investigated the roles of miR-34 in axotomy-induced axon degeneration in OSNs and γ axon pruning in MB neurons. We found that ectopically overexpressed miR-34 impaired γ axon pruning in differentiated MB neurons, but did not inhibit axon degeneration in OSNs following axotomy. Notably, ectopic miR-34 overexpression downregulated the expression of EcR-B1 in MB γ neurons, and restoration of the EcR-B1 expression through over-expressing Babo and EcR-B1 rescued the miR-34-induced axon pruning defect in the MB neurons. Our findings regarding how miRNAs regulate developmentally related large-scale axon degeneration in *Drosophila* provide new insight into the regulatory mechanisms involved in nervous system development.

## Results

### Ectopic overexpression of miR-34 impairs the mushroom body (MB) γ lobe pruning

**Figure 1:**
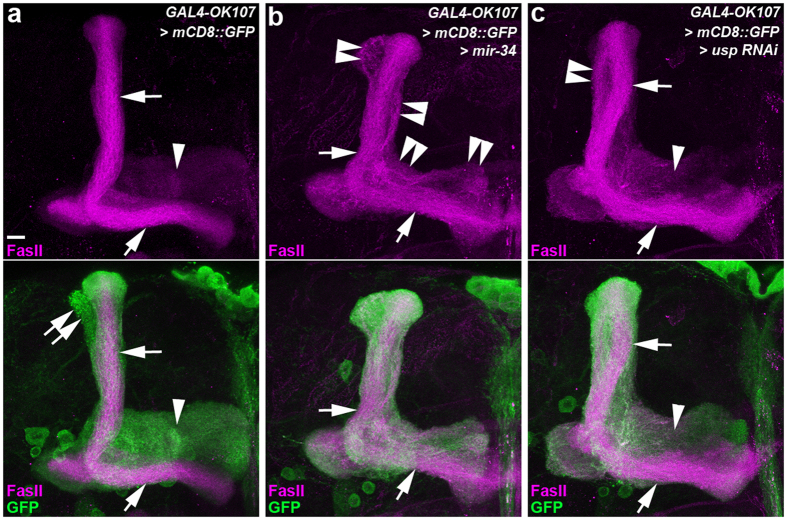
Ectopic miR-34 overexpression caused aberrant lobe formation on mushroom body (MB) neurons. Confocal images show the lobe phenotype of mushroom body (MB) neurons in wild-type flies (**a**) and flies with ectopic miR-34 overexpression (**b**) and RNAi knockdown of USP (**c**). Fasciculin II (FasII) staining (magenta) reveals the dorsal α and medial β lobes (arrows, strong magenta staining) and the medial γ lobes (arrowheads, faint magenta staining) on MB neurons. In the lower panels, GAL4-OK107-driven mCD8::GFP (GFP) expression (green) shows the morphology of α lobes (arrows), α′ lobes (double arrows), and γ lobes (arrowheads). (**a–c**) Compared to the α and β lobes of wild-type MB neurons (arrows, a), ectopic miR-34 expression resulted in aberrant axonal branches projecting adjacent to the α and β lobes (double arrowheads, **b**). This miR-34 induced lobe defect is similar to that of the γ lobe phenotype observed in MB neurons in which USP expression was knocked down by RNAi (double arrowhead, **c**). Fly genotypes are listed in Supplemental Table 2. Scale bar: 10 μm for all panels.

Because the adult-onset elevation of miR-34 expression plays an essential role in inhibiting chronic neural deterioration in the aged brain^7^, we investigated whether miR-34 also functions in the inhibition of axotomy-induced axon degeneration in OSNs or developmentally related γ axon pruning in MB neurons. We removed the antennae and maxillary palps of flies, thereby separating OSN axons from their soma, to establish a system for examining whether miR-34 influences axon degeneration following axotomy, as previously described^10^. We found that, in wild-type OSNs and those in which miR-34 was ectopically overexpressed, degeneration was induced 3 days following axotomy (Supplemental Figure 1), suggesting that miR-34 does not protect against axotomy-induced axon degeneration in OSNs. On the other hand, using the pan MB neuronal driver, GAL4-OK107 that expresses GAL4 in MB α/β, α′/β′ and γ neurons^21^, the ectopic overexpression of miR-34 caused defects in the γ lobe formation in which excessive axonal branches often projected adjacent to the MB α and β lobes (Fig. 1a,b, Supplemental Table 1). This defect in the MB lobe formation was similar to that in a previous report of defective γ axon pruning in MB neurons expressing LOF *usp* mutants^19^ (Fig. 1c, Supplemental Table 1). These results collectively suggested that miR-34 overexpression may disrupt axon pruning in MB γ neurons.

**Figure 2:**
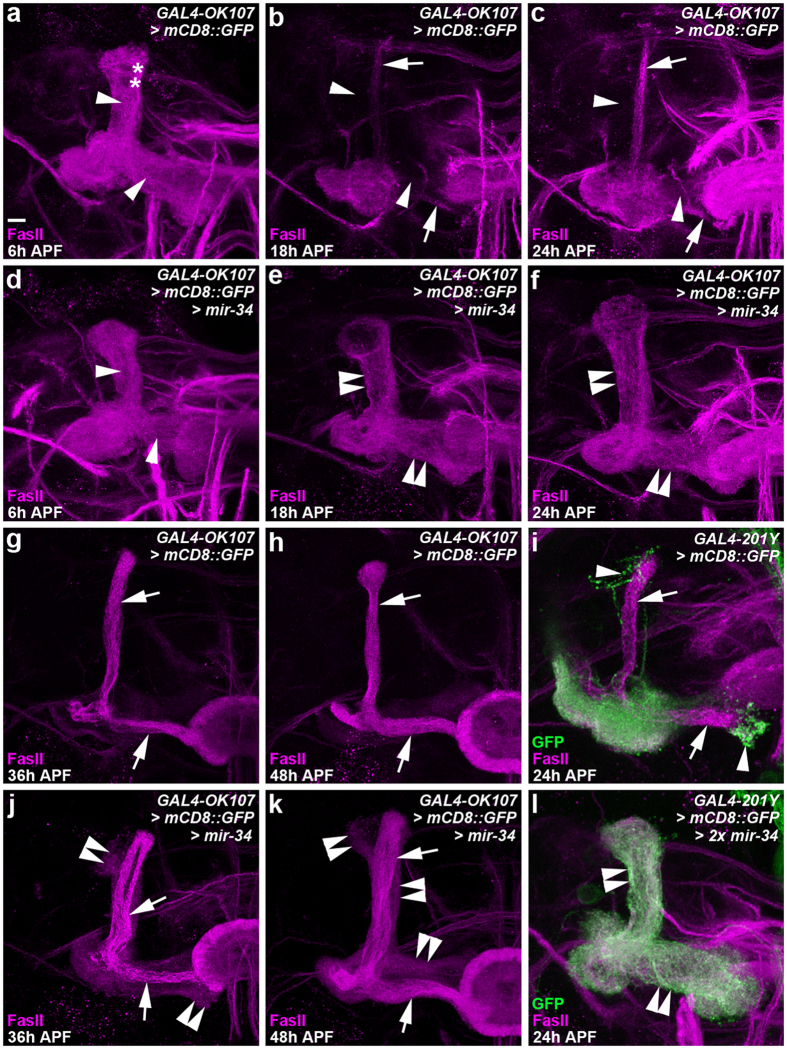
Ectopic miR-34 overexpression inhibited γ lobe pruning in MB neurons. Confocal images show γ lobes of MB neurons in wild-type flies (**a–c**,**g–i**) and flies that were for ectopic miR-34 overexpression (one copy of miR-34 in (**d–f**,**j**,**k)**; two copies of miR-34 in l) driven by GAL4-OK107 (**a–h**,**j**,**k**) or GAL4-201Y (**i** and **l**). FasII staining (magenta) and mCD8::GFP (GFP) expression (green) of MB neurons reveal the morphology of the larval-specific γ lobes (arrowheads) and α and β lobes (arrows) at various time points after puparium formation (APF). In MB neurons of wild-type flies (**a–c** and **g–i**), the process of pruning larval-specific γ lobes (arrowheads) appeared to be initiated in the γ lobes themselves, where the lack of FasII staining was observed from 6 h APF (asterisks, a) to the near completion of axon pruning at 18 h APF (arrowheads). The developing α and β lobes were observed from 24 h APF onward (arrows, **c**,**g–i**). By contrast, in the MB neurons with ectopic miR-34 overexpression, a significant fraction of larval-specific γ lobes was remained at 18–24 h APF (double-arrowheads, **e,f,i**), and aberrant axonal braches (most likely γ lobe-derived) were located adjacent to the developing α and β lobes at 36-48 h APF (double-arrowheads, **j,k**). Fly genotypes are listed in Supplemental Table 2. Scale bar: 10 μm for all panels.

To investigate whether the underlying mechanism of the miR-34-mediated perturbation of MB lobe formation was similar to that involved in axon pruning in MB γ neurons, we compared the morphogenesis of γ lobes in wild-type MB neurons with that of MB neurons in which miR-34 was ectopically overexpressed at various time points from 6 to 48 h after puparium formation (APF; Fig. 2, Supplemental Table 1). Axon pruning was initiated in wild-type MB neurons at 6 h APF, and the disappearance of larval-specific γ lobes was observed at 18 h APF (Fig. 2a,b). By contrast, a substantial fraction of larval-specific γ lobes was preserved at 24 h APF in MB neurons in which miR-34 ectopic overexpression was driven by GAL4-OK107 (Fig. 2d–f). The aberrant larval-specific γ lobes persisted at 36 and 48 h APF in flies with ectopically overexpressed miR-34, but not in wild-type flies (Fig. 2g,h,j,k). The formation of aberrant larval-specific γ lobes was also observed in MB neurons in which miR-34 was ectopically overexpressed using GAL4-201Y (Fig. 2i,l, Supplemental Table 1), which expresses GAL4 in differentiated MB γ neurons and a small subset of core MB α/β neurons^18^. We were, however, unable to examine the effect of GAL4-201Y-driven miR-34 expression on MB γ lobe pruning beyond the mid-pupal stage due to toxicity related to the ectopic overexpression of miR-34 (data not shown). These results collectively suggested that the overexpression of miR-34 impaired axon pruning in MB γ neurons.

### Ectopic miR-34 overexpression in differentiated MB γ neurons causes aberrant axon pruning

Because the GAL4-OK107 expresses GAL4 in MB progenitor cells, including neuroblasts and ganglion mother cells, as well as the MB γ, α′/β′ and α/β neuronal types^21^,^22^, we speculated that the effect of ectopically overexpressed miR-34 on γ lobe pruning in MB neurons might be caused by a miR-34-induced change in the developmental fate of the MB progenitor cells that resulted in the elimination of axon pruning, rather than the direct inhibition of axon pruning in differentiated MB γ neurons. To investigate this possibility, we used Asense-GAL4, which expresses GAL4 in neural progenitors and young neurons but not in differentiated neurons^23^, to drive the expression of miR-34. No defect in γ lobe pruning was observed in MB neurons derived from Asense-GAL4^+^ progenitor cells and young neurons in which miR-34 was overexpressed (Supplemental Figure 2, Supplemental Table 1). We also used Asense-GAL80 in separate experiments to suppress GAL4-OK107 driven miR-34 overexpression in neural progenitors and young neurons, but not in differentiated MB neurons^24^, and this manipulation cannot inhibit the miR-34-induced γ lobe pruning defect (Supplemental Figure 3, Supplemental Table 1). These results suggested that the miR-34-induced impairment of γ axon pruning in MB neurons was caused by miR-34 overexpression in differentiated MB neurons, and was not the result of altered developmental fate due to miR-34 overexpression in progenitor cells and young neurons.

**Figure 3:**
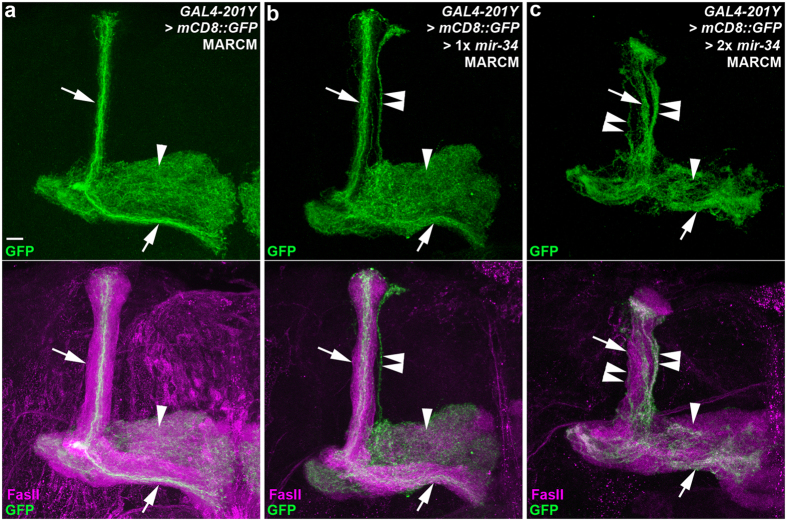
Ectopic miR-34 overexpression in differentiated MB neurons disrupted γ axon pruning. Confocal images show axon pruning of MB neurons in wild-type flies (**a**) and flies with ectopic miR-34 overexpression using the mosaic analysis with a repressible cell marker system (**b**,**c**). GAL4-201Y-driven mCD8::GFP (GFP) expression (green) reveals the morphology of γ lobes (arrowheads) and a subset of α and β lobes (arrows). In the lower panels, FasII staining (magenta) reveals the α and β lobes (strong magenta staining; arrows) and the medial γ lobes (faint magenta staining; arrowheads). A subset of the dorsal α lobe was observed on MB neurons in wild-type flies (upper arrow, **a**), whereas an aberrant axonal bundle (likely unpruned γ lobe) projected outside the MB α lobe (double arrowhead, **b**) and most of the γ lobes were intact in the sample with ectopic expression of one copy of *mir-34* transgene (arrowhead, **b**). Defective γ lobe pruning was more severe in flies for ectopic overexpression of two copies of miR-34 transgenes in MB γ neurons (double arrowheads, **c**). Fly genotypes are listed in Supplemental Table 2. Scale bar: 10 μm for all panels.

Because the defective MB γ lobe pruning phenotype was induced by miR-34 overexpression in nearly all differentiated MB neurons, we speculated whether a cell-autonomous mechanism was involved in the defective axon pruning phenotype. To investigate this possibility, we employed the mosaic analysis with a repressible cell marker (MARCM) system to evaluate the effect of ectopic miR-34 overexpression using GAL4-201Y, which expresses GAL4 in fully differentiated MB γ neurons and a small subset of core MB α/β neurons^18^,^25^ (Fig. 3a–c, Supplemental Table 1). Using this strategy in differentiated MB γ neurons, we observed that the effect of ectopically overexpressed miR-34 on the defective γ lobe pruning phenotype was dose-dependent, in which one copy of miR-34 exhibited a moderate, less-penetrant phenotype (80%, n = 5; Fig. 3b, Supplemental Table 1) and two copies of miR-34 overexpression exhibited a severe, fully-penetrant phenotype (100%, n = 13; Fig. 3c, Supplemental Table 1). These results combined with those of the analysis of stage-specific miR-34 overexpression suggested that the induction of the miR-34-mediated perturbation of γ axon pruning occurred after the differentiation of MB γ neurons.

### Ectopic miR-34 overexpression downregulates the endogenous EcR-B1 expression in MB neurons exhibiting defective γ lobe pruning

**Figure 4:**
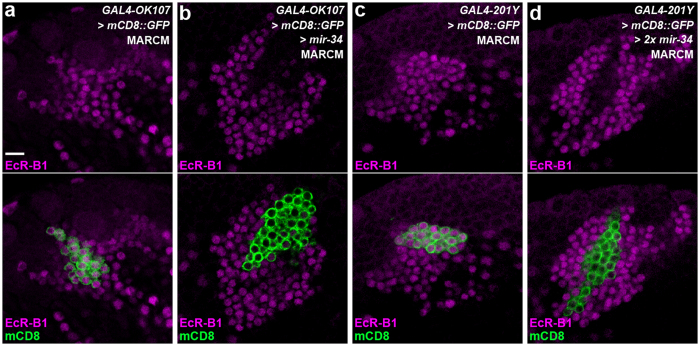
Ectopic miR-34 overexpression downregulated EcR-B1 expression in MB neurons. Confocal images show EcR-B1 expression in MB neurons in wild-type flies (**a**,**c**) and flies for ectopic miR-34 overexpression (one copy of miR-34 in **b**; two copies of miR-34 in **d**) using the mosaic analysis with a repressible cell marker (MARCM) system. The MARCM clones were induced as newly hatched larvae, and the brains were dissected at the wandering larval stage (WL) or the white pupal stage (WP). In the lower panels, the expression of mCD8::GFP (green) driven by GAL4-OK107 (**a**,**b**) or GAL4-201Y (**c**,d) outlines the cell bodies of MARCM clones, whereas EcR-B1 staining (magenta) reveals ecdysone-responsive neurons. The expression of EcR-B1 was significantly reduced by ectopic miR-34 overexpression in the MARCM clones (**b,d**), compared to that of the wild-type MARCM clones (**a,c**). Fly genotypes are listed in Supplemental Table 2. Scale bar: 10 μm for all panels.

**Figure 5:**
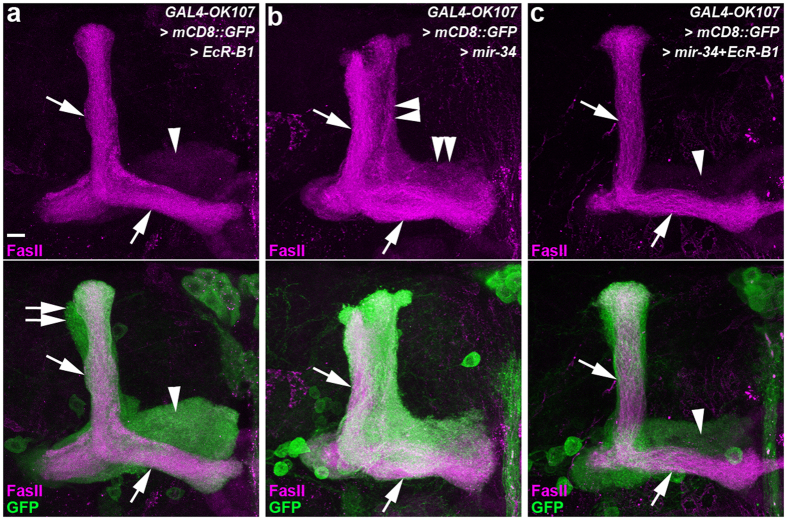
Overexpression of EcR-B1 rescued the miR-34-induced defective γ lobe phenotype in MB neurons. Confocal images show the lobe pruning phenotypes of MB neurons in flies with ectopic overexpression of EcR-B1 (**a**), ectopic overexpression of miR-34 (**b**), or the overexpression of both (**c**). FasII staining reveals the medial γ lobes (arrowheads, faint magenta staining) and dorsal α and medial β lobes (arrows, strong magenta staining). In the lower panels, GAL4-OK107-driven mCD8::GFP (GFP) expression (green) shows the morphology of α lobes (arrows), α′ lobes (double arrows), and γ lobes (arrowheads). Overexpression of EcR-B1 in MB neurons had no observable effect on the MB lobe pruning phenotype (**a**). Ectopic miR-34 overexpression in MB neurons caused defective γ lobe pruning (double-arrowheads, **b**). The defective γ lobe pruning phenotype was rescued by overexpression of EcR-B1 in MB neurons with ectopic miR-34 overexpression (**c**). Fly genotypes are listed in Supplemental Table 2. Scale bar: 10 μm for all panels.

**Figure 6:**
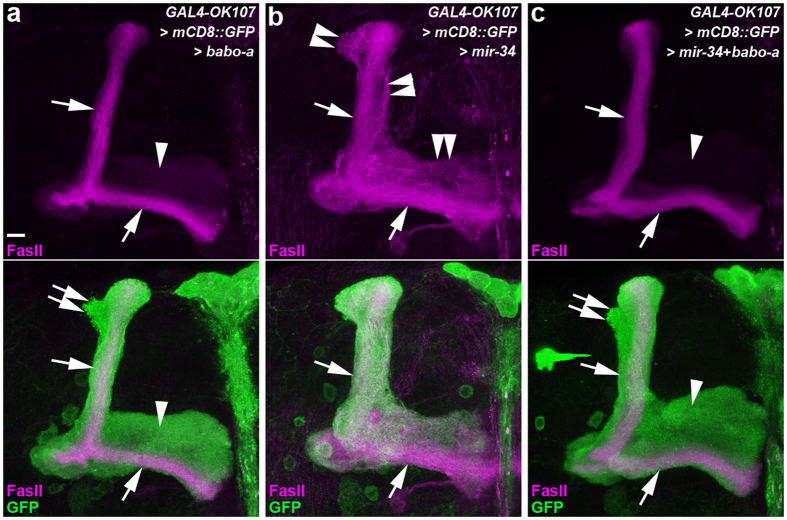
Overexpression of Babo-a rescued the miR-34-induced defective γ lobe phenotype in MB neurons. Confocal images show lobe pruning phenotypes of MB neurons in flies with ectopic overexpression of Babo-a (**a**), ectopic overexpression of miR-34 (**b**), or the overexpression of both (**c**). FasII staining reveals the medial γ lobes (arrowheads, faint magenta staining) and dorsal α and medial β lobes (arrows, strong magenta staining). In the lower panels, GAL4-OK107-driven mCD8::GFP (green) shows the morphology of α lobes (arrows), α′ lobes (double arrows), and γ lobes (arrowheads). Overexpression of Babo-a in MB neurons had no observable effect on the MB lobe pruning phenotype (**a**). Ectopic miR-34 overexpression in MB neurons caused defective γ lobe pruning (double-arrowheads, **b**). The defective γ lobe pruning phenotype was rescued by overexpression of Babo-a in MB neurons with ectopic miR-34 overexpression (**c**). Fly genotypes are listed in Supplemental Table 2. Scale bar: 10 μm for all panels.

Previous studies reported that MB γ axon pruning defects in *babo* and *smox* mutants occurred through the downregulation of EcR-B1 expression^19^,^20^. Therefore, we investigated whether ectopically expressed miR-34 also reduces the expression of EcR-B1 in MB neurons that in turn elicits the defective γ lobe pruning phenotype. To test this hypothesis, we applied the MARCM system to ectopically express miR-34 using GAL4-OK107 and GAL4-201Y in MB neurons in separate experiments. We found that EcR-B1 expression in MARCM clones was substantially lower than that in the wild-type MARCM clones, and no obvious difference in EcR-B1 expression between the wild-type control MARCM clones and non-MARCM neurons was observed (n = 4 for all experiments; Fig. 4a–d). Notably, we also found that overexpression of EcR-B1 or Babo-a, but not the β-galactosidase control, rescued miR-34-induced defective γ lobe pruning in MB neurons, which suggested that miR-34 may regulate EcR-B1 expression via the Babo/EcR signaling pathway (Figs 5 and 6, Supplemental Table 1; data not shown, n = 20 for the β-galactosidase control). In additional experiments, the overexpression of EcR-B1 or the Babo isoform, Babo-a, alone had no observable effect on the γ lobe phenotype (Figs 5a and 6a). These results collectively suggested that ectopic overexpression of miR-34 results in defective axon pruning in MB γ neurons through the downregulation of EcR-B1 expression.

### RNAi knockdown of Eip74EF, Hr4, and yem does not affect miR-34-induced defective γ lobe pruning in MB neurons

Although the results of our experiments suggested that miR-34 influenced γ axon pruning via the downregulation of EcR-B1 expression, it remained unclear whether other miR-34-regulated genes play essential roles in controlling axon pruning in MB γ neurons. Using a web-based genome-wide prediction algorithm ( http://www.microrna.org/), we identified Eip74EF, hormone receptor 4 (Hr4), and yemanuclein (yem) as mRNAs possessing 3′ untranslated regions (UTRs) with miR-34 target sites (Supplemental Figure 4a). Among these, Eip74EF has previously been shown to be an miR-34 target that functions in the regulation of neurodegeneration^7^, and all three genes are annotated as having transcription factor and DNA binding activities. We confirmed that the 3′ UTRs of Eip74EF, Hr4, and yem contain functional miR-34 target sites using a previously described luciferase reporter gene assay^26^ (Supplemental Figure 4b). However, RNA interference (RNAi) experiments in which the expression of each transcript was knocked down separately, defective γ lobe pruning in MB neurons was not observed (Supplemental Figure 4c–f). These results indicate that neither Eip74EF, Hr4, nor yem alone is crucial for γ axon pruning in MB γ neurons. Thus, it remains unclear whether the expression of proteins involved in γ lobe pruning, other than EcR-B1, are also influenced by the level of miR-34 expression.

## Discussion

Throughout development and during adulthood, the dendrites and axons of neurons in the CNS exhibit a range of processes in which these projections may undergo elaboration, growth, or elimination in order to establish connections with other neurons along defined neural paths. Understanding how these reorganizational processes are regulated in neurons may provide insight into the mechanisms by which these neural connections are lost in neurodegenerative diseases. In *Drosophila*, axon pruning in MB γ neurons is a developmentally related type of large-scale axon degeneration in which MB γ neurons remodel larval-specific dorsal and medial axon branches and produce adult-specific medial axon branches^18^.

We investigated the role of *Drosophila* miR-34 in γ axon pruning in MB neurons to identify pathways that contribute to axon remodeling in developing MB neurons. Using an experimental approach in which miR-34 was ectopically expressed, we found that miR-34, which is known to protect against chronic neurodegeneration in the aged *Drosophila* brain, can inhibit developmentally related axon pruning in MB γ neurons (Fig. 2b), but cannot inhibit axotomy-induced axon degeneration in OSN axons (Supplemental Figure 1). Our investigation showed that miR-34 mediates defects in γ lobe pruning in differentiated MB neurons via the downregulation of EcR-B1 expression in MB γ neurons (Figs 3, 4 and 5), and that the overexpression of Babo-a rescued the miR-34-induced γ lobe pruning defect in MB neurons (Fig. 6). However, we cannot confirm whether babo is a direct target of miR-34 since we observed no obvious change in Babo expression in wild-type, miR-34 overexpression and *mir-34* mutant flies based on western blot analysis using an anti-Babo antibody (ab14682; Abcam; Lai, *et al*., unpublished observation). Previous studies have shown that the expression of EcR-B1 in MB γ neurons is enhanced upon the binding of the glial-cell-derived TGF-β homolog, Myoglianin (Myo), to Babo, and the Babo signaling can be also facilitated by the presence of an immunoglobulin superfamily protein, Plum^20^,^27^,^28^. Our finding that the overexpression of Babo-a reversed the effect of ectopically overexpressed miR-34 on the γ lobe pruning defect suggests that miR-34 may be possible to regulate the expression of Myo and Plum that lie upstream of Babo-a^20^,^27^,^28^. It is also possible that miR-34 regulated the expression of EcR-B1 through pathways that function parallel to Babo-a, such as TGF-β-independent Fushi tarazu transcription factor 1 (Ftz-f1) and Hormone receptor-like in 39 (Hr39) mediated signaling pathways, which have also been shown to contribute to axon pruning in MB γ neurons^29^. Future studies are warranted to examine the effects of miR-34 on the regulatory influence of Myo, Plum, Ftz-f1 and Hr39 on EcR-B1 expression.

We also used a computational strategy to perform a genome-wide search of genes possessing 3' UTRs with miRNA target sites, in which the Eip74EF, Hr4, and yem transcripts were identified as potential miR-34 targets (Supplemental Figure 4a,b). However, the individual RNAi-based knockdown of these transcripts had no obvious γ lobe pruning defect in MB neurons (Supplemental Figure 4c–f). It is possible that γ axon pruning in MB neurons might be mediated by a combination of Eip74EF, Hr4, yem, and/or other yet-to-be-identified miR-34 target genes. Future investigations are warranted to identify and characterize other miR-34 target genes that might contribute to the regulation of EcR-B1 expression in axon pruning in MB γ neurons to obtain further insight into the regulation of developmentally related large-scale axon degeneration.

## Methods

### Production of *UAS-mir-34, UAS-Eip74EF RNAi*, and *UAS-yem RNAi* transgenic flies

Standard molecular biological techniques were used to generate the *UAS-mir-34, UAS-Eip74EF RNAi*, and *UAS-yem RNAi* transgenes. Primers for the amplification of sequences of *mir-34* (cagtttccaccgcacttttgttc and cccttaaatattccctcttggc), *Eip74EF* (gattgcttgacaataggaattt and tatttaccgtcgatttagctgg), and *yem* (gttaaactcgggatacttgttc and agttttcattatatgcggatcg) were designed using a previously described method^30^. The production of transgenic flies carrying the *UAS-mir-34, UAS-Eip74EF RNAi* and *UAS-yem RNAi* transgenes was performed by BestGene (Chino Hills, CA, USA).

### Fly strains

The following fly strains used in this study: (1) *UAS-mCD8::GFP* on the second chromosome^25^; (2) *Or88a-GAL4*^31^; (3) *GAL4-OK107*^21^; (4) *UAS-mir-34*^*[1]*^; (5) *UAS-mir-34*^*[2]*^; (6) *UAS-usp RNAi*^32^; (7) *hs-FLP*^*[122]*^^33^; (8) *FRT*^*[G13]*^*,UAS-mCD8::GFP,GAL4–201Y*^18^; (9) *FRT*^*[82B]*^*,tubP-GAL80*^25^; (10) *FRT*^*[82B]*^^34^; (11) *yw,hs-FLP*^*[1]*^*,UAS-mCD8::GFP*^25^; (12) *UAS-EcR-B1*^20^; (13) *UAS-baba-a*^35^; (14) *UAS-lacZ.NZ* (Bloomington stock (BL) 3956); (15) *Asense-GAL4*^23^; (16) *Asense-GAL80*^24^; (17) *UAS-Eip74EF RNAi*; (18) *UAS-Hr4 RNAi* (BL 31868); (19) *UAS-yem RNAi*.

### Generation of the samples with OSN axotomy and the MARCM clones

In the OSN axotomy experiments, the antennae and maxillary palps were removed from flies in order to separate OSN axons from their soma^10^. The axon-severed flies were kept at 25 °C for one, three and five days before the brain dissection. Mosaic clones for the MARCM studies were generated, as previously described^25^. In short, the MARCM samples were obtained by collecting embryos in vials, and inducing mosaic clones by heat-shocking newly hatching larvae for 10 to 20 min. The MARCM flies were kept at 25 °C until reaching the developmental stage desired for brain dissection.

### Fly brain preparation and image processing

Dissection, immunostaining, and mounting of fly brains were performed as described previously^25^. For primary antibodies, rabbit anti-GFP (1:800) was purchased from Life Technologies (Grand Island, NY, USA), whereas mouse anti-Fasciculin II (1D4, 1:50) and mouse anti-EcR-B1 (AD4.4, 1:50) were purchased from Developmental studies Hybridoma Bank (Iowa City, IA, USA). Secondary antibodies conjugated to Alexa 488, 546, or 647 (1:800) were purchased from Life Technologies. Immunofluorescent images were recorded by using a Zeiss LSM 700 or 780 confocal microscope (Oberkochen, Germany), and were processed using the Zeiss LSM image browser for projecting images from confocal stacks and Photoshop computer software (Adobe, San Jose, CA, USA) for adjusting image intensity only.

### Luciferase reporter gene Assay

The luciferase reporter gene assay was conducted using the Dual-Glo Luciferase Assay System (Promega, Madison, WI, USA) according to an optimized version of the manufacturer’s protocol. The 3′ UTR regions of *Eip74EF, Hr4*, and *yem* were inserted following the luciferase coding region. miR-1 was transfected as a negative control for the miR-34 experiments.

## Additional Information

**How to cite this article**: Lai, Y.-W. *et al.* *Drosophila* microRNA-34 Impairs Axon Pruning of Mushroom Body g Neurons by Downregulating the Expression of Ecdysone Receptor. *Sci. Rep.* **6**, 39141; doi: 10.1038/srep39141 (2016).

**Publisher's note:** Springer Nature remains neutral with regard to jurisdictional claims in published maps and institutional affiliations.

## Supplementary Material

Supplementary Information

## References

[b1] Kandel E. R. Principles of neural science. 5th edn, (McGraw-Hill, 2013).

[b2] Im H. I. & Kenny P. J. MicroRNAs in neuronal function and dysfunction. Trends Neurosci 35, 325–334, doi: 10.1016/j.tins.2012.01.004 (2012). 22436491 PMC3565236

[b3] Chen W. & Qin C. General hallmarks of microRNAs in brain evolution and development. RNA Biol 12, 701–708, doi: 10.1080/15476286.2015.1048954 (2015). 26000728 PMC4615839

[b4] Cao D. D., Li L. & Chan W. Y. MicroRNAs: Key Regulators in the Central Nervous System and Their Implication in Neurological Diseases. Int J Mol Sci 17, doi: 10.3390/ijms17060842 (2016). PMC4926376 27240359

[b5] Ballatore C., Lee V. M. & Trojanowski J. Q. Tau-mediated neurodegeneration in Alzheimer’s disease and related disorders. Nat Rev Neurosci 8, 663–672, doi: 10.1038/nrn2194 (2007). 17684513

[b6] Dickson J. R., Kruse C., Montagna D. R., Finsen B. & Wolfe M. S. Alternative polyadenylation and miR-34 family members regulate tau expression. J Neurochem 127, 739–749, doi: 10.1111/jnc.12437 (2013). 24032460 PMC3859707

[b7] Liu N. . The microRNA miR-34 modulates ageing and neurodegeneration in Drosophila. Nature 482, 519–523, doi: 10.1038/nature10810 (2012). 22343898 PMC3326599

[b8] Neukomm L. J. & Freeman M. R. Diverse cellular and molecular modes of axon degeneration. Trends Cell Biol 24, 515–523, doi: 10.1016/j.tcb.2014.04.003 (2014). 24780172 PMC4149811

[b9] Yaniv S. P. & Schuldiner O. A fly’s view of neuronal remodeling. Wiley Interdiscip Rev Dev Biol 5, 618–635, doi: 10.1002/wdev.241 (2016). 27351747 PMC5086085

[b10] Osterloh J. M. . dSarm/Sarm1 is required for activation of an injury-induced axon death pathway. Science 337, 481–484, doi: 10.1126/science.1223899 (2012). 22678360 PMC5225956

[b11] George E. B., Glass J. D. & Griffin J. W. Axotomy-induced axonal degeneration is mediated by calcium influx through ion-specific channels. J Neurosci 15, 6445–6452 (1995). 7472407 10.1523/JNEUROSCI.15-10-06445.1995 PMC6577979

[b12] Chuang C. F. & Bargmann C. I. A Toll-interleukin 1 repeat protein at the synapse specifies asymmetric odorant receptor expression via ASK1 MAPKKK signaling. Genes Dev 19, 270–281, doi: 10.1101/gad.1276505 (2005). 15625192 PMC545892

[b13] Avery M. A. . WldS prevents axon degeneration through increased mitochondrial flux and enhanced mitochondrial Ca2+ buffering. Curr Biol 22, 596–600, doi: 10.1016/j.cub.2012.02.043 (2012). 22425157 PMC4175988

[b14] Xiong X. . The Highwire ubiquitin ligase promotes axonal degeneration by tuning levels of Nmnat protein. PLoS Biol 10, e1001440, doi: 10.1371/journal.pbio.1001440 (2012). 23226106 PMC3514318

[b15] Perry V. H., Brown M. C. & Lunn E. R. Very Slow Retrograde and Wallerian Degeneration in the CNS of C57BL/Ola Mice. Eur J Neurosci 3, 102–105 (1991). 12106273 10.1111/j.1460-9568.1991.tb00815.x

[b16] Mack T. G. . Wallerian degeneration of injured axons and synapses is delayed by a Ube4b/Nmnat chimeric gene. Nat Neurosci 4, 1199–1206, doi: 10.1038/nn770 (2001). 11770485

[b17] Sasaki Y. & Milbrandt J. Axonal degeneration is blocked by nicotinamide mononucleotide adenylyltransferase (Nmnat) protein transduction into transected axons. J Biol Chem 285, 41211–41215, doi: 10.1074/jbc.C110.193904 (2010). 21071441 PMC3009846

[b18] Lee T., Lee A. & Luo L. Development of the Drosophila mushroom bodies: sequential generation of three distinct types of neurons from a neuroblast. Development 126, 4065–4076 (1999). 10457015 10.1242/dev.126.18.4065

[b19] Lee T., Marticke S., Sung C., Robinow S. & Luo L. Cell-autonomous requirement of the USP/EcR-B ecdysone receptor for mushroom body neuronal remodeling in Drosophila. Neuron 28, 807–818, doi: 10.1016/S0896-6273(00)00155-0 (2000). 11163268

[b20] Zheng X. . TGF-beta signaling activates steroid hormone receptor expression during neuronal remodeling in the Drosophila brain. Cell 112, 303–315 (2003). 12581521 10.1016/s0092-8674(03)00072-2

[b21] Connolly J. B. . Associative learning disrupted by impaired Gs signaling in Drosophila mushroom bodies. Science 274, 2104–2107 (1996). 8953046 10.1126/science.274.5295.2104

[b22] Zhan X. L. . Analysis of Dscam diversity in regulating axon guidance in Drosophila mushroom bodies. Neuron 43, 673–686, doi: 10.1016/j.neuron.2004.07.020 (2004). 15339649

[b23] Zhu S. . Gradients of the Drosophila Chinmo BTB-zinc finger protein govern neuronal temporal identity. Cell 127, 409–422, doi: 10.1016/j.cell.2006.08.045 (2006). 17055440

[b24] Neumuller R. A. . Genome-wide analysis of self-renewal in Drosophila neural stem cells by transgenic RNAi. Cell Stem Cell 8, 580–593, doi: 10.1016/j.stem.2011.02.022 (2011). 21549331 PMC3093620

[b25] Lee T. & Luo L. Mosaic analysis with a repressible cell marker for studies of gene function in neuronal morphogenesis. Neuron 22, 451–461, doi: 10.1016/S0896-6273(00)80701-1 (1999). 10197526

[b26] Jin Y., Chen Z., Liu X. & Zhou X. Evaluating the microRNA targeting sites by luciferase reporter gene assay. Methods Mol Biol 936, 117–127, doi: 10.1007/978-1-62703-083-0_10 (2013). 23007504 PMC3646406

[b27] Awasaki T., Huang Y., O’Connor M. B. & Lee T. Glia instruct developmental neuronal remodeling through TGF-beta signaling. Nat Neurosci 14, 821–823, doi: 10.1038/nn.2833 (2011). 21685919 PMC3337551

[b28] Yu X. M. . Plum, an immunoglobulin superfamily protein, regulates axon pruning by facilitating TGF-beta signaling. Neuron 78, 456–468, doi: 10.1016/j.neuron.2013.03.004 (2013). 23664613 PMC3706783

[b29] Boulanger A. . ftz-f1 and Hr39 opposing roles on EcR expression during Drosophila mushroom body neuron remodeling. Nat Neurosci 14, 37–44, doi: 10.1038/nn.2700 (2011). 21131955

[b30] Chen C. H. . A synthetic maternal-effect selfish genetic element drives population replacement in Drosophila. Science 316, 597–600, doi: 10.1126/science. 1138595 (2007). 17395794

[b31] Couto A., Alenius M. & Dickson B. J. Molecular, anatomical, and functional organization of the Drosophila olfactory system. Curr Biol 15, 1535–1547, doi: 10.1016/j.cub.2005.07.034 (2005). 16139208

[b32] Lin S., Huang Y. & Lee T. Nuclear receptor unfulfilled regulates axonal guidance and cell identity of Drosophila mushroom body neurons. PLoS One 4, e8392, doi: 10.1371/journal.pone.0008392 (2009). 20027309 PMC2793019

[b33] Marin E. C., Watts R. J., Tanaka N. K., Ito K. & Luo L. Developmentally programmed remodeling of the Drosophila olfactory circuit. Development 132, 725–737, doi: 10.1242/dev.01614 (2005). 15659487

[b34] Xu T. & Rubin G. M. Analysis of genetic mosaics in developing and adult Drosophila tissues. Development 117, 1223–1237 (1993). 8404527 10.1242/dev.117.4.1223

[b35] Ting C. Y. . Photoreceptor-derived activin promotes dendritic termination and restricts the receptive fields of first-order interneurons in Drosophila. Neuron 81, 830–846, doi: 10.1016/j.neuron.2013.12.012 (2014). 24462039 PMC4004379

