# Supplementary material for: Drosophila microRNA-34 Impairs Axon Pruning of Mushroom Body γ Neurons by Downregulating the Expression of Ecdysone Receptor
Source: Sci Rep. 2016 Dec 23;6:39141. doi: 10.1038/srep39141 (PMC5180235; doi:10.1038/srep39141)
Supplement: Supplementary Information [file srep39141-s1.pdf]

Drosophila microRNA-34 Impairs Axon Pruning of Mushroom Body γ Neurons by Downregulating the Expression of Ecdysone Receptor

Yen-Wei Lai[1,2,3], Sao-Yu Chu[1], Jia-Yi Wei[1], Chu-Ya Cheng[2], Jian-Chiuan Li[2], Chun-Hong Chen[2,3]*

and Hung–Hsiang Yu[1]*

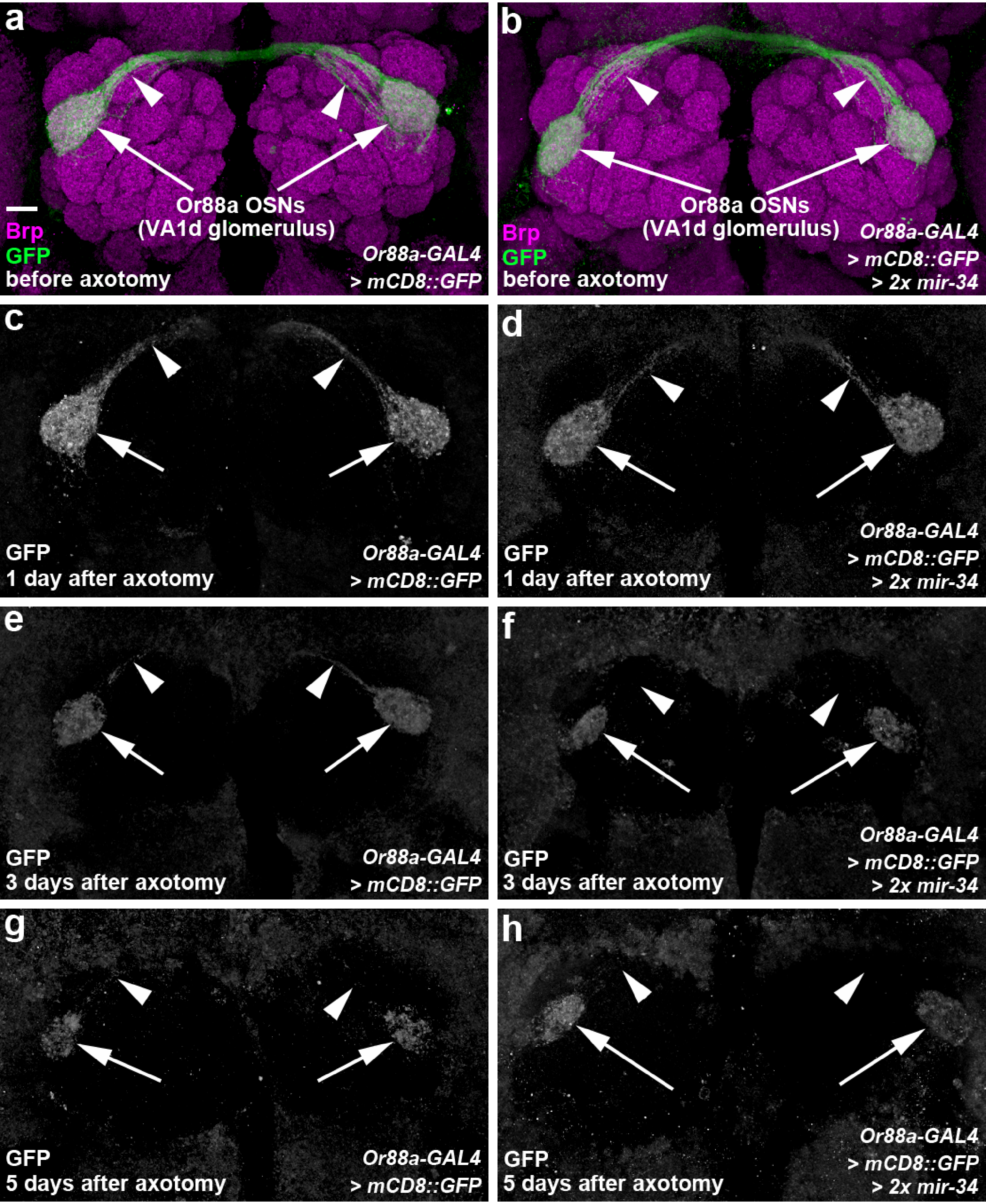
a
Or88a OSNs
(VA1d glomerulus)
Brp
GFP
before axotomy
Or88a-GAL4
> mCD8::GFP
b
Or88a OSNs
(VA1d glomerulus)
Brp
GFP
before axotomy
Or88a-GAL4
> mCD8::GFP
> 2x mir-34
c
GFP
1 day after axotomy
Or88a-GAL4
> mCD8::GFP
d
GFP
1 day after axotomy
Or88a-GAL4
> mCD8::GFP
> 2x mir-34
e
GFP
3 days after axotomy
Or88a-GAL4
> mCD8::GFP
f
GFP
3 days after axotomy
Or88a-GAL4
> mCD8::GFP
> 2x mir-34
g
GFP
5 days after axotomy
Or88a-GAL4
> mCD8::GFP
h
GFP
5 days after axotomy
Or88a-GAL4
> mCD8::GFP
> 2x mir-34

Supplemental Figure 1. Ectopic miR-34 overexpression in olfactory sensory neurons (OSNs) did not prevent axotomy-induced axon degeneration. Confocal images show the axon degeneration phenotype of Or88a-expressing OSNs in wild-type flies (a, c, e, and g) and flies with ectopic miR-34 overexpression (b, d, f, and h) before axotomy (a [n=8] and b [n=9]) and at 1 day (c [n=16] and d [n=14]), 3 days (e [n=8] and f [n=8]), and 5 days (g [n=8] and h [n=8]) after axotomy. Bruchpilot staining (Brp; magenta, a and b) reveals the structure of the antennal lobes, and Or88a-GAL4-driven mCD8::GFP (green in a and b, or white in c-h) reveals the axonal nerves (arrows) and axonal terminals (arrowheads; innervating the VA1d glomerulus) of Or88a-expressing OSNs. (a-h) Ectopic miR-34 overexpression in Or88a-expressing OSNs did not inhibit axotomy-induced axon degeneration, as evidenced by the similarity in clearance of the axonal nerves and terminals observed in Or88a-expressing OSNs in both wild-type flies and flies with ectopic miR-34 overexpression. Fly genotypes are listed in Supplemental Table 2. Scale bar: 10 μm for all panels.

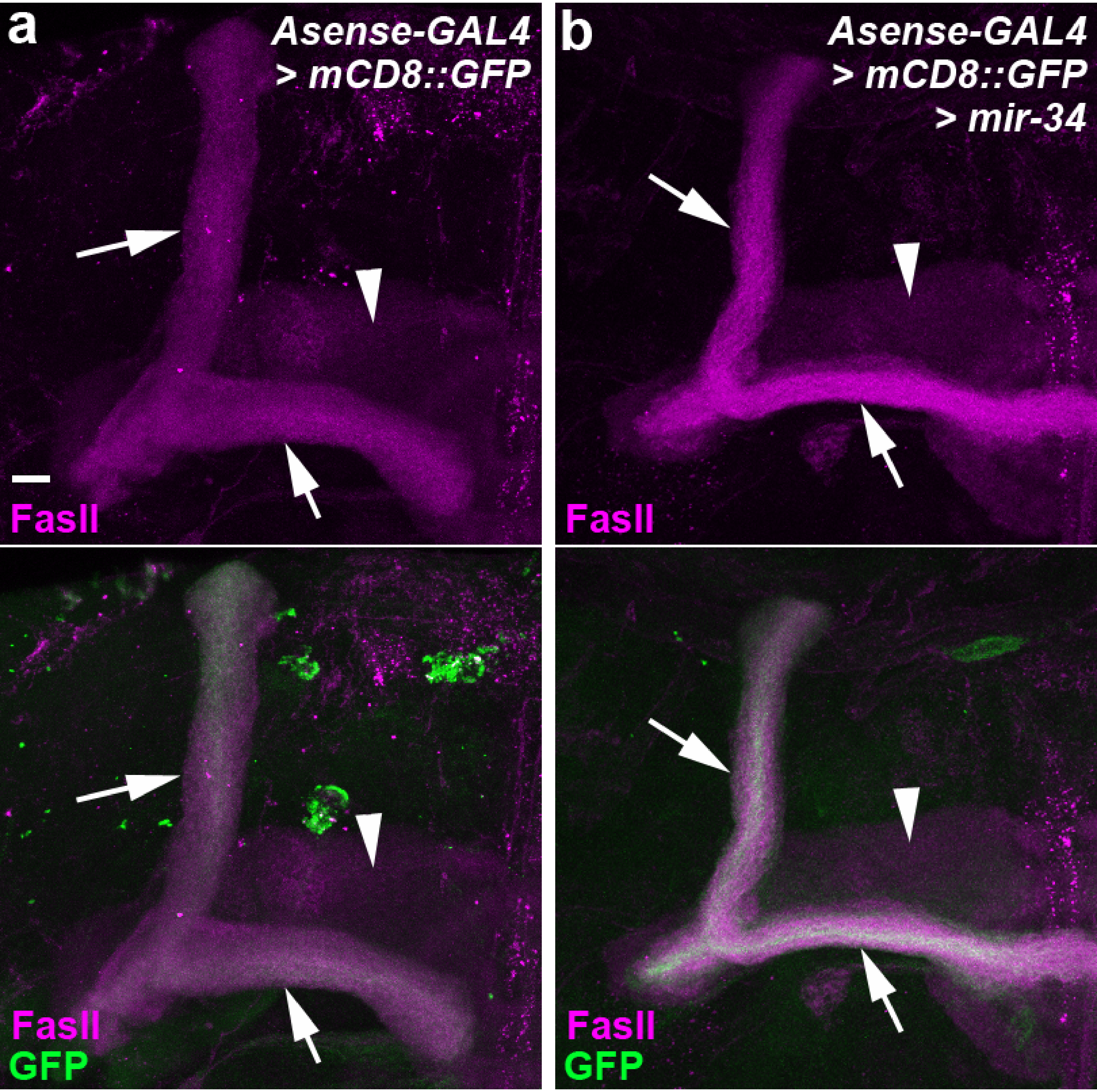
a
Asense-GAL4
> mCD8::GFP
FasII
FasII
GFP
b
Asense-GAL4
> mCD8::GFP
> mir-34
FasII
FasII
GFP

Supplemental Figure 2. Ectopic miR-34 overexpression in neural progenitor cells and young neurons did not cause defective γ lobe pruning in mushroom body (MB) neurons. Confocal images show the lobe pruning phenotype of MB neurons in wild-type flies (a) and flies with MB neurons descended from lineages in which GAL4-OK107-driven ectopic overexpression of miR-34 was restricted to neural progenitor cells and young neurons using Asense-GAL4 (b). Fasciculin II (FasII) staining (magenta) reveals the dorsal α and medial β lobes (arrows, strong magenta staining) and the medial γ lobes (arrowheads, faint magenta staining) of MB neurons. In the lower panels, mCD8::GFP (GFP) expression (green) in core α and β lobes suggest the persistence of late-stage MB α/β neurons. (a an d b) Similar to the MB neurons of wild-type flies, ectopic miR-34 overexpression restricted to neural progenitor cells and young neurons did not disrupt γ lobe pruning in MB neurons. Fly genotypes are listed in Supplemental Table 2. Scale bar: 10 μm for all panels.

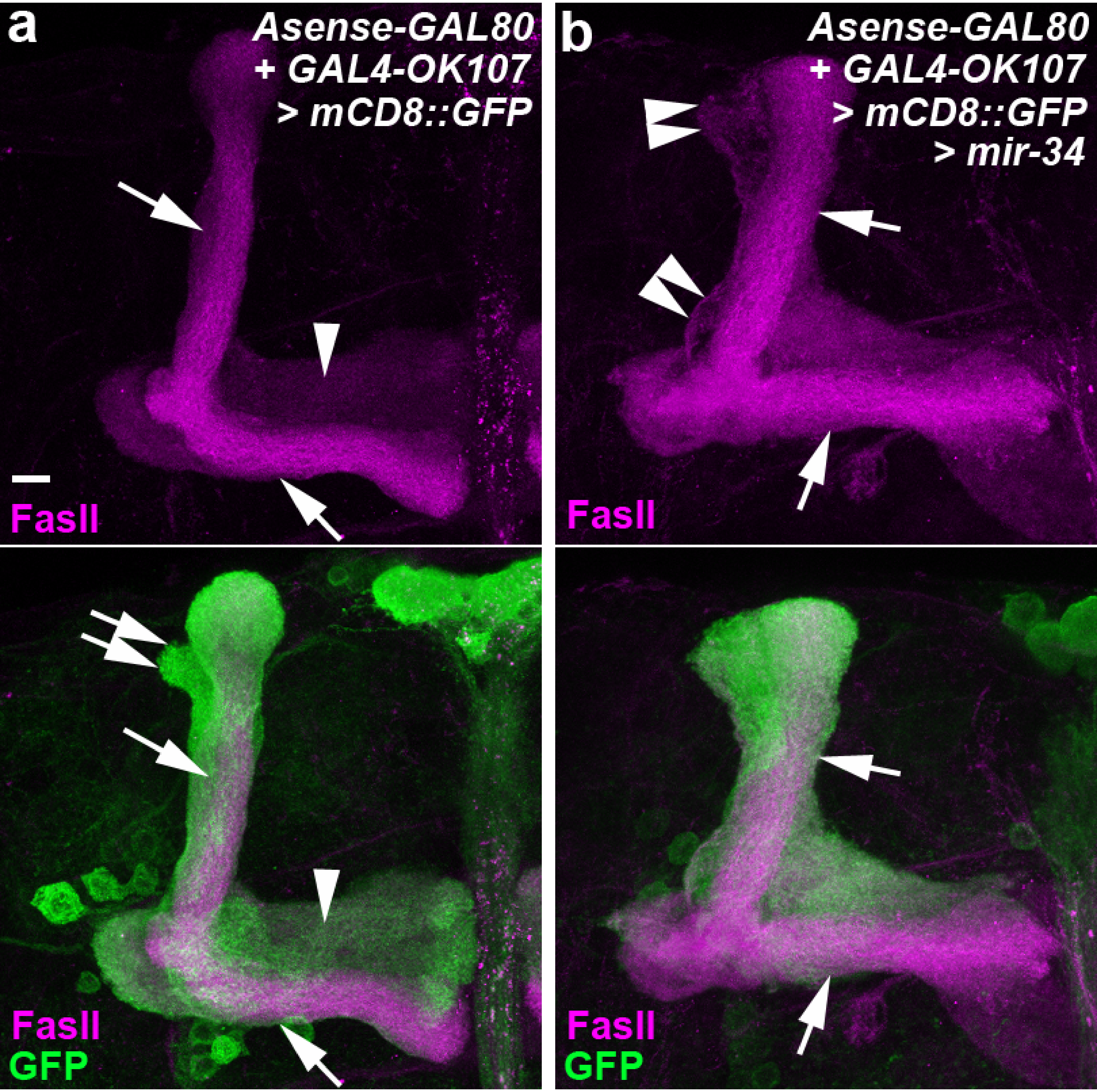
a
Asense-GAL80
+ GAL4-OK107
> mCD8::GFP
FasII
b
Asense-GAL80
+ GAL4-OK107
> mCD8::GFP
> mir-34
FasII
FasII
GFP
FasII
GFP

Supplemental Figure 3. Ectopic miR-34 overexpression in differentiated MB neurons caused defective γ lobe pruning. Confocal images show the lobe pruning phenotype of MB neurons in wild-type flies (a) and flies in which miR-34 was ectopically overexpressed in differentiated neurons by using GAL4-OK107 and Asense-GAL80 (b). FasII staining (magenta) reveals the dorsal α and medial β lobes (arrows, strong magenta staining) and the medial γ lobes (arrowheads, faint magenta staining) of MB neurons. In the lower panels, GAL4-OK107-driven mCD8::GFP (GFP) expression (green) reveals the morphology of α lobes (arrows), α' lobes (double arrows), and γ lobes (arrowheads). (a and b) In contrast to the MB neurons in wild-type flies (a), the differentiated MB neurons in flies with ectopic miR-34 overexpression exhibited defective γ lobe pruning (double arrowheads, b). Fly genotypes are listed in Supplemental Table 2. Scale bar: 10 μm for all panels.

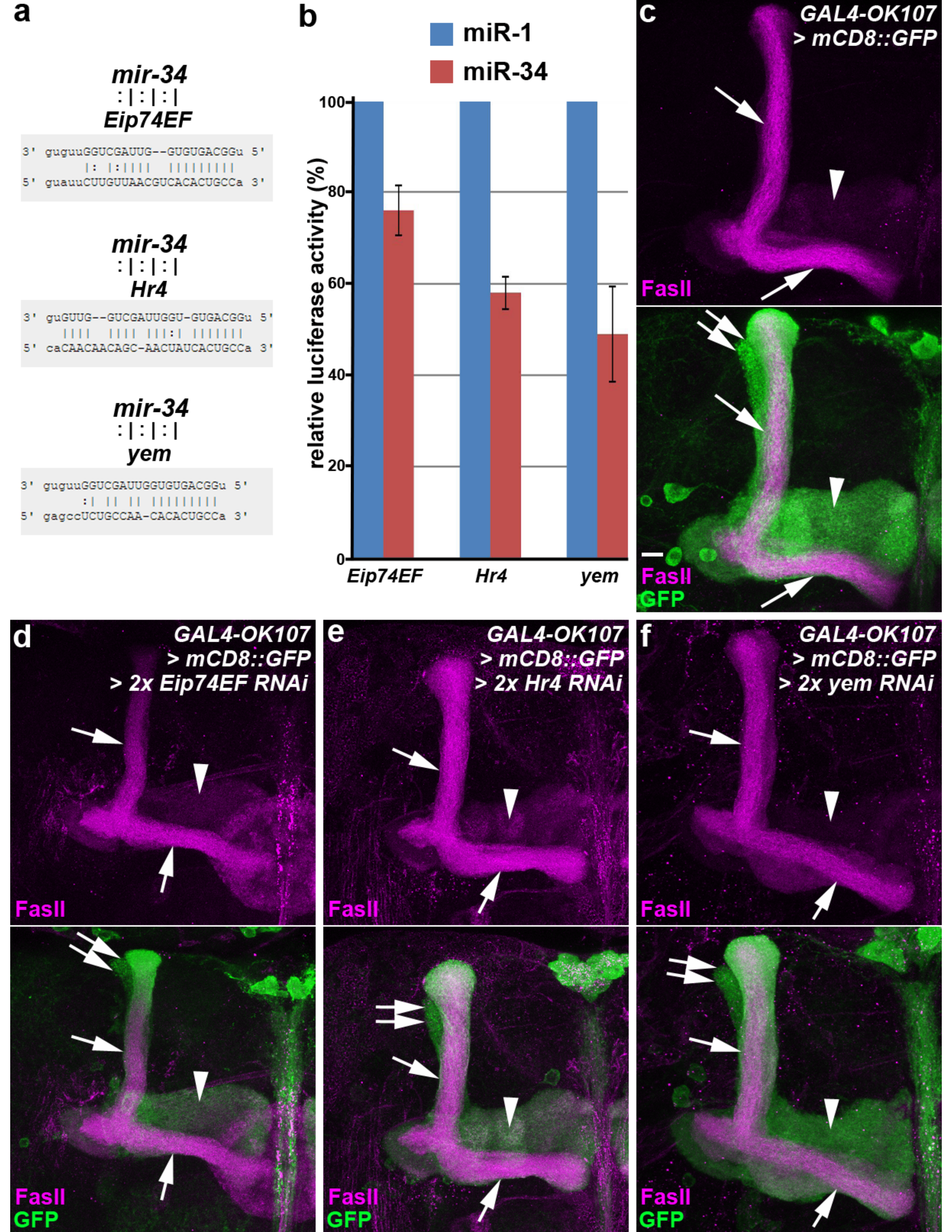
a
mir-34
Eip74EF
3' guguuGGUCGAUUG--GUGUGACGGu 5'
5' guauuCUUGUUAACGUCACACUGCCa 3'
mir-34
Hr4
3' guGUUG--GUCGAUUGGU-GUGACGGu 5'
5' caCAACAACAGC-AACUAUCACUGCCa 3'
mir-34
yem
3' guguuGGUCGAUUGGUGUGACGGu 5'
5' gagccUCUGCCAA-CACACUGCCa 3'
b
miR-1
miR-34
relative luciferase activity (%)
100
80
60
40
20
0
Eip74EF
Hr4
yem
c
GAL4-OK107
> mCD8::GFP
FasII
FasII
GFP
d
GAL4-OK107
> mCD8::GFP
> 2x Eip74EF RNAi
FasII
FasII
GFP
e
GAL4-OK107
> mCD8::GFP
> 2x Hr4 RNAi
FasII
FasII
GFP
f
GAL4-OK107
> mCD8::GFP
> 2x yem RNAi
FasII
FasII
GFP

Supplemental Figure 4. Separate knockdown of Eip74EF, Hr4 and yem expression using RNAi did not affect γ lobe pruning. (a) The Eip74EF, Hr4 and yem transcripts contain multiple miR-34 target sites. The highest-score for alignment with the miR-34 target sequence is shown. (b) A luciferase reporter gene assay was used to confirm that miR-34 silenced the expression of Eip74EF, Hr4, and yem, and miR-1 was used to normalize the relative luciferase activity for miR-34. (c-f) Confocal images show the lobe pruning phenotype of MB neurons in wild-type flies (c) and flies in which the expression of the Eip74EF, Hr4, and yem mRNA were knocked down separately using RNAi (d-f). FasII staining (magenta) reveals dorsal α and medial β lobes (arrows, strong magenta staining) and the medial γ lobes (arrowheads, faint magenta staining) of MB neurons. In the lower panels, GAL4-OK107-driven mCD8::GFP (GFP) expression (green) reveals the morphology of α lobe (arrows), α' lobes (double arrows), γ lobes (arrowheads). Similar to the MB neurons in the wild-type flies (c), defective γ lobe pruning was not observed in MB neurons in which Eip74EF, Hr4, and yem was knocked down using RNAi. Fly genotypes are listed in Supplemental Table 2. Scale bar: 10 μm for panels c-f.

Supplemental Table 1. Frequencies of the γ lobe pruning phenotype of mushroom body neurons in the flies shown in the figures

Manipulation (developmental stage analyzed)	Total examined (n)	Defective γ lobe pruning rate	Figure
GAL4-OK107>mCD8::GFP (6h AFP)	10	0%	2a
GAL4-OK107>mCD8::GFP (18h AFP)	10	0%	2b
GAL4-OK107>mCD8::GFP (24h AFP)	10	0%	2c
GAL4-OK107>mCD8::GFP (36h AFP)	14	0%	2g
GAL4-OK107>mCD8::GFP (48h AFP)	16	0%	2h
GAL4-OK107>mCD8::GFP (adult)	20	0%	1a, S4c
GAL4-OK107>mCD8::GFP>1x mir-34 (6h APF)	10	0%	2d
GAL4-OK107>mCD8::GFP>1x mir-34 (18h APF)	10	100%	2e
GAL4-OK107>mCD8::GFP>1x mir-34 (24h APF)	20	90%	2f
GAL4-OK107>mCD8::GFP>1x mir-34 (36h APF)	12	100%	2j
GAL4-OK107>mCD8::GFP>1x mir-34 (48h APF)	14	100%	2k
GAL4-OK107>mCD8::GFP>1x mir-34 (adult)	20	100%	1b, 5b, 6b
GAL4-OK107>mCD8::GFP>usp RNAi (adult)	20	100%	1c
GAL4-OK107>mCD8::GFP>EcR-B1 (adult)	14	0%	5a
GAL4-OK107>mCD8::GFP>1x mir-34>EcR-B1 (adult)	20	10%	5c
GAL4-OK107>mCD8::GFP>babo-a (adult)	16	0%	6a
GAL4-OK107>mCD8::GFP>1x mir-34>EcR-B1 (adult)	16	0%	6c
GAL4-OK107>mCD8::GFP>2x Eip74EF RNAi (adult)	6	0%	S4d
GAL4-OK107>mCD8::GFP>2x Hr4 RNAi (adult)	4	0%	S4e
GAL4-OK107>mCD8::GFP>2x yem RNAi (adult)	6	0%	S4f
GAL4-201Y>mCD8::GFP (24h APF)	20	0%	2i
GAL4-201Y>mCD8::GFP>2x mir-34 (24h APF)	20	100%	2l
GAL4-201Y>mCD8::GFP (adult; MARCM)	14	0%	3a
GAL4-201Y>mCD8::GFP>1x mir-34 (adult; MARCM)	5	80%	3b
GAL4-201Y>mCD8::GFP>2x mir-34 (adult; MARCM)	13	100%	3c
Asense-GAL4>mCD8::GFP (adult)	10	0%	S2a
Asense-GAL4>mCD8::GFP>1x mir-34 (adult)	6	0%	S2b
Asense-GAL80+GAL4-Ok107>mCD8::GFP (adult)	12	0%	S3a
Asense-GAL80+GAL4-Ok107>mCD8::GFP>1x mir-34 (adult)	20	100%	S3b

APF, after puparium formation

Supplemental Table 2. Genotypes of the flies in the figures

**Figure**	**Genotype**
1a, 2a-c, 2g, 2h, S4c	*yw;UAS-mCD8::GFP/+;+;GAL4-OK107/+*
1b, 2d−f, 2j, 2k, 5b, 6b	*yw;UAS-mCD8::GFP/UAS-mir-34[1];+;GAL4-OK107/+*
2i	*yw;FRT*^{G13}*,UAS-mCD8::GFP,GAL4-201Y /+;+;+*
2l	*yw;FRT*^{G13}*,UAS-mCD8::GFP,GAL4-201Y / UAS-mir-34[1],UAS-mir-34[2];+;+*
1c	*yw;UAS-mCD8::GFP/UAS-usp RNAi;+;GAL4-OK107/+*
3a, 4c	*yw,hs-FLP[122]/yw;FRT*^{G13}*,UAS-mCD8::GFP,GAL4-201Y/+;FRT*^{82B}*, tubP-GAL80/FRT*^{82B}
3b	*yw,hs-FLP[122]/yw;FRT*^{G13}*,UAS-mCD8::GFP,GAL4-201Y/UAS-mir-34[1];FRT*^{82B}*, tubP-GAL80/FRT*^{82B}
3c, 4d	*yw,hs-FLP[122]/yw;FRT*^{G13}*,UAS-mCD8::GFP,GAL4-201Y/UAS-mir-34[1],UAS-mir-34[2]; FRT*^{82B}*,tubP-GAL80/FRT*^{82B}
4a	*yw,hs-FLP[1],UAS-mCD8::GFP/yw;+;FRT*^{82B}*,tubP-GAL80/FRT*^{82B}*;GAL4-OK107/+*
4b	*yw,hs-FLP[1],UAS-mCD8::GFP/yw;UAS-mir-34[1]/+;FRT*^{82B}*,tubP-GAL80/ FRT*^{82B}*; GAL4-OK107/+*
5a	*yw;UAS-mCD8::GFP/+;UAS-EcR-B1/+;GAL4-OK107/+*
5c	*yw;UAS-mCD8::GFP/UAS-mir-34[1];UAS-EcR-B1/+;GAL4-OK107/+*
6a	*yw,UAS-baba-a /yw;UAS-mCD8::GFP/+;+;GAL4-OK107/+*
6c	*yw,UAS-baba-a/yw;UAS-mCD8::GFP/UAS-mir-34[1];+; GAL4-OK107/+*
S1a, S1c, S1e, S1g	*yw;UAS-mCD8::GFP/+;Or88a-GAL4/+;+*
S1b, S1d, S1f, S1h	*yw;UAS-mCD8::GFP/ UAS-mir-34[1],UAS-mir-34[2];Or88a-GAL4/+;+*
S2a	*yw;Asense-GAL4,UAS-mCD8::GFP/+;+;+*
S2b	*yw;Asense-GAL4,UAS-mCD8::GFP/UAS-mir-34[1];+;+*
S3a	*yw;UAS-mCD8::GFP/+;Asense-GAL80/+;GAL4-OK107/+*
S3b	*yw;UAS-mCD8::GFP/ UAS-mir-34[1];Asense-GAL80/+;GAL4-OK107/+*
S4d	*yw;UAS-mCD8::GFP/+;UAS-Eip74EF RNAi/UAS-Eip74EF RNAi;GAL4-OK107/+*
S4e	*yw;UAS-mCD8::GFP/+;UAS-yem RNAi/UAS-yem RNAi;GAL4-OK107/+*
S4f	*yw;UAS-mCD8::GFP/+;UAS-Hr4 RNAi/UAS-Hr4 RNAi;GAL4-OK107/+*
